# Evolution of an Expanded Mannose Receptor Gene Family

**DOI:** 10.1371/journal.pone.0110330

**Published:** 2014-11-12

**Authors:** Karen Staines, Lawrence G. Hunt, John R. Young, Colin Butter

**Affiliations:** The Pirbright Institute, Compton, United Kingdom; CSIRO, Australia

## Abstract

Sequences of peptides from a protein specifically immunoprecipitated by an antibody, KUL01, that recognises chicken macrophages, identified a homologue of the mammalian mannose receptor, MRC1, which we called MRC1L-B. Inspection of the genomic environment of the chicken gene revealed an array of five paralogous genes, *MRC1L-A* to *MRC1L-E*, located between conserved flanking genes found either side of the single *MRC1* gene in mammals. Transcripts of all five genes were detected in RNA from a macrophage cell line and other RNAs, whose sequences allowed the precise definition of spliced exons, confirming or correcting existing bioinformatic annotation. The confirmed gene structures were used to locate orthologues of all five genes in the genomes of two other avian species and of the painted turtle, all with intact coding sequences. The lizard genome had only three genes, one orthologue of *MRC1L-A* and two orthologues of *the MRC1L-B* antigen gene resulting from a recent duplication. The Xenopus genome, like that of most mammals, had only a single *MRC1*-like gene at the corresponding locus. *MRC1L-A* and *MRC1L-B* genes had similar cytoplasmic regions that may be indicative of similar subcellular migration and functions. Cytoplasmic regions of the other three genes were very divergent, possibly indicating the evolution of a new functional repertoire for this family of molecules, which might include novel interactions with pathogens.

## Introduction

Recent evolution of the repertoire of molecules involved in the function of the immune system has resulted in substantial divergence in the composition and functions of the gene families to which these molecules belong. Even among mammals, different families of molecules may carry out equivalent functions in different species [1]. While the functions of many molecules in immunity are well conserved between mammalian and avian species, in other cases there is extensive divergence in molecular repertoires, with cytokines and chemokines providing examples [2]. These differences often involve gene duplication followed by functional diversification [3]. Thus evolution has led to variety in molecular details in spite of more conserved underlying mechanisms in solutions to the problems of infection. Variation in molecular repertoires may underlie some of the differences between species in host-pathogen interactions. An understanding of these differences will be essential to optimise approaches to immune protection.

The mannose receptor C-type 1 gene (*MRC1, CD206*) is the eponymous member of the mannose receptor family. Their gene products are type I transmembrane glycoproteins containing arrays of C-type lectin domains (CTLDs). The family also includes DEC205 (CD205), MRC2 (Endo180, CD280) and Phospholipase A_2_ receptor (PLA_2_R), each having important functions in immunity [4]. These receptors all have an N-terminal cysteine-rich domain (CysR) followed by a single fibronectin type II domain (FNII), then either 8 (MRC1, MRC2 and PLA_2_R) or 10 (DEC205) CTLDs separated by linker regions. They have a transmembrane domain and a short cytoplasmic tail containing motifs that signal endocytosis. In mammals, *DEC205* and *PLA_2_R* genes are arranged in tandem on one chromosome, while the others are unlinked. In the three genes encoding 8 CTLDs, the 30 exon gene structure and the splicing phases of all introns are completely conserved. The CTLDs fall into two groups, one having an extra pair of cysteine residues at the N-terminal end of the domain (domains 2, 3, 4, 6, 8) [5]. While individual CTLDs generally have low affinities for carbohydrate ligands, the molecules can exhibit high affinities for complex carbohydrate by cooperative binding [6]. Only the fourth CTLD of human MRC1 retains strong enough binding to have lectin activity on its own [7].

The mannose receptor is a recycling endocytosis receptor, rapidly internalised via clathrin-coated vesicles and delivered to early endosomes, with the majority of the receptors in the intracellular location in the steady state [8]. Endocytosis of bound molecules underlies the primary function of the mannose receptor in the recognition of pathogen associated molecular patterns and their consequent uptake for engulfment and for antigen presentation [9]. A soluble form of the mammalian mannose receptor, produced by proteolytic cleavage [10], may also function in the delivery of antigens to lymphoid follicles [11]. Clearance by binding to the mannose receptor may also be involved in the regulation of levels of some hormones [12]. In chickens, the orthologue of mammalian PLA_2_R acts as an Fc receptor (FcRY), the functional equivalent of mammalian FcRn, extending the range of its endocytic targets to immune complexes [13].

Binding of the mannose receptor by a virus may elicit immunomodulatory responses [14]. It may also facilitate viral entry in a cell either indirectly, as with HIV [15], or directly, as with Dengue [16]. In the mouse, binding of influenza virus by the mannose receptor, in addition to its more widespread binding to sialic acid, is important for virus entry into macrophages [17]. The virus replicates inside infected macropahges, but they do not release infective virus. Instead, the infection enhances the presentation of influenza virus antigens and stimulates the generation of pro-inflammatory cytokines. Thus the participation of the mannose receptor in allowing infection of macrophages contributes to innate and eventually to adaptive protection [18]. Reciprocally driven evolution of the virus and the mannose receptor in different species may thus be a significant contributor to differences in host-pathogen interactions.

Employing mass spectrometry of immunoprecipitated antigen, we identified a molecule recognised by a macrophage marker antibody, KUL01 [19], as a chicken homologue of MRC1. Inspection of neighbouring avian genome sequence revealed that the locus contained five tandemly repeated genes encoding similar molecules that are likely to have arisen through duplication, of a single ancestral MRC1 gene, in the avian lineage. Very different cytoplasmic sequences and differences in relative transcript levels in tissues indicate diversification of function among the duplicated genes.

## Results

### The KUL01 antibody recognises a homologue of the macrophage mannose receptor MRC1

**Figure 1:**
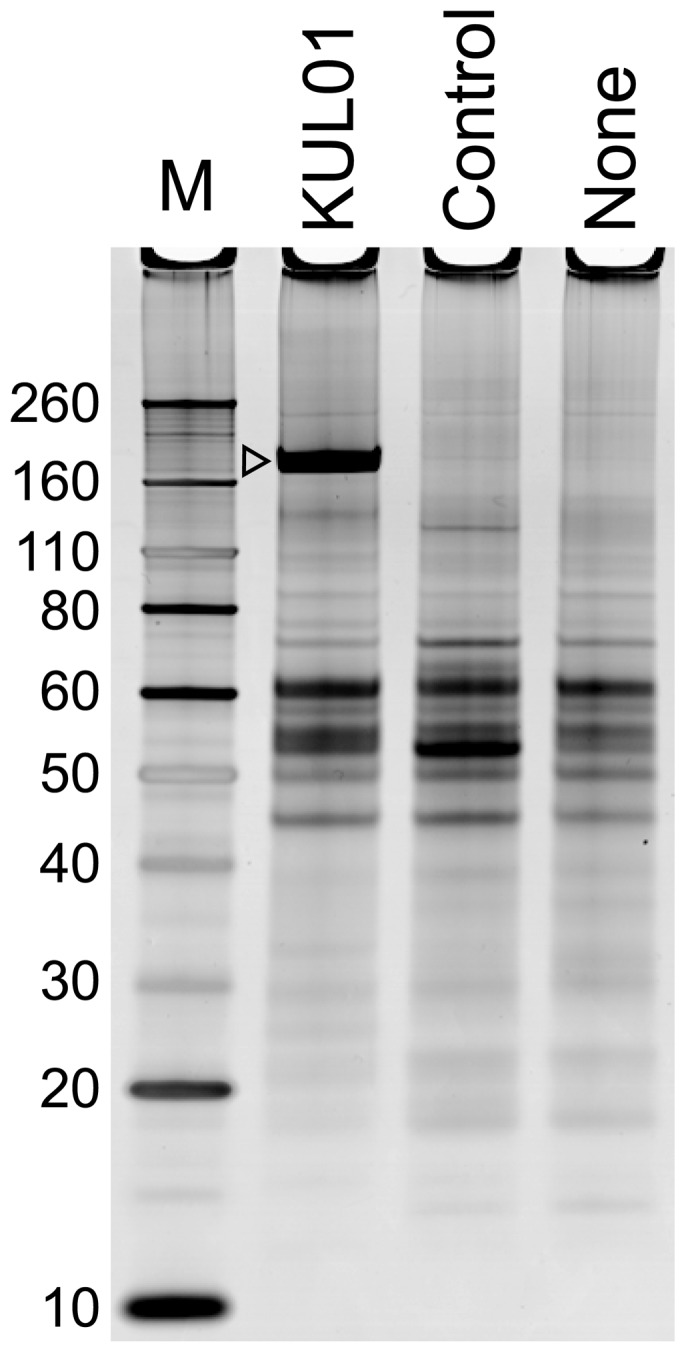
KUL01 specifically precipitates a molecule with apparent molecular weight 180 kDa. Track M contains molecular weight standards. The other tracks contain materials absorbed from a precleared lysate of the HD11 macrophage cell line, by agarose beads to which were attached either KUL01, an isotype matched control antibody, or no antibody, and eluted at low pH. The open arrowhead points to the band(s) specifically absorbed by the KUL01 antibody, which were analysed by mass spectroscopy.

KUL01 antibody bound to agarose beads was used to adsorb proteins from a lysate of the transformed chicken macrophage cell line HD11, which were analysed by SDS PAGE after elution at low pH. Specific bands, obtained from beads coated with KUL01 but not from those coated with control antibody, were excised, digested with trypsin and analysed my mass spectroscopy. The major specifically recognised molecule was a (doublet) band with an apparent molecular weight of 180 kDa (figure 1). By Mascot search of the NCBI non-redundant chicken proteins in the IPI database, a sufficient number of peptides from the tryptic digest of this band were identified as being derivable from the sequence IPI00814304 to unequivocally identify it as the source of antigen specifically adsorbed by KUL01 (figure S1 and table S1). It was annotated as being a chicken homologue of MRC1.

### Genomic context of chicken MRC1 orthologues

The genomic context of the gene for the KUL01 antigen was inspected to see whether additional evidence from conserved gene order would support its identification as the orthologue of MRC1. Inspection of the region between orthologues of the highly conserved genes, *SLC39A12* and *STAM*, that flank *MRC1* in mammals, revealed multiple segments with similarity to the *MRC1* gene. Existing annotation and EST data, together with manual examination, allowed the definition of five potential *MRC1L* genes. For convenience, these were labelled *MRC1L-A to E* in sequence in the direction of their transcription (which is inverse to the genome map). Annotations of this gene array from different sources varied widely, in detailed exon composition, splicing sites and numbers of genes. To evaluate the predicted gene models, a series of PCR primers were designed for amplification of segments of the predicted transcripts from RNA. PCR products were amplified from RNA from the HD11 transformed macrophage cell line, and from a cDNA library from RPRL Line 0 chicken spleen.

**Figure 2:**
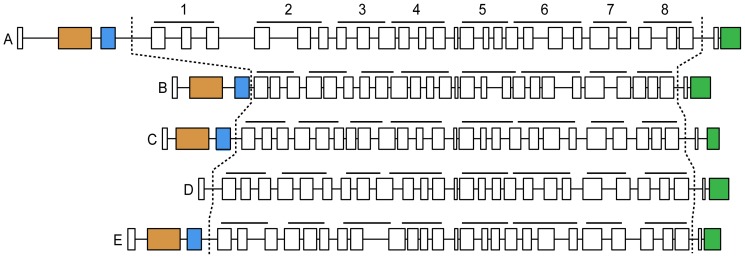
Structure of paralogous *MRC1* genes in the chicken genome. Exons are shown to scale as rectangles. Introns are drawn to 1/10 of the exon scale, except for the shortest which are expanded for visibility. Orange and blue exons are the CysR and FNII domains in all genes except D. The terminal green exon contains transmembrane and cytoplasmic regions. The central array of exons encodes the eight CTLDs indicated by the black bars above each gene.

All predicted exons were amplified from spliced transcripts from the Line 0 chicken cDNA. All the transcript sequences confirmed in this way contained intact reading frames for MRC1-like proteins. These were submitted to the ENA database and received accession numbers HF569039, HF566127, HF569040, HF569041, HF569042, in order *MRC1L-(A to E)* and are provided in figure S2, together with their genomic locations. The *MRC1L-B* and *–C* genes are now correctly annotated in the ENSEMBL database (ENSGALT00000043091, ENSGALT00000014059). Annotation of the other genes is currently inaccurate, with errors as described in file S1 and are liable to change in subsequent database versions. Differences from the corresponding red jungle fowl genomic sequences are enumerated in table S2. The exon structures of the genes and their coding content are compared in figure 2. All encoded eight CTLDs. All except D also contained the exons encoding CysR and FNII receptor domains. That exception apart, the 30-exon structures are identical to that of the mammalian MRC1 genes, with all splice phases conserved and very similar exon lengths for all except the terminal exons.

One alternative splice acceptor site, for exon 8 of the MRC1L-E gene, resulting in the insertion of six amino acids, was found in a minority of the sequenced clones from Line 0 cDNA. While that was the only variant transcript in the Line 0 cDNAs, in the HD11 RNA, more frequent alternatively spliced transcripts were detected for *MRC1L-E*, most of which resulted in interruption of the open reading frame, so that no intact open reading frame for *MRC1L-E* was found in the HD11 cDNA. Thus it is possible the alternative splicing seen in HD11 was an artefact of the transformation of these cells. The alternative spliced transcripts are illustrated in figure S3.

**Figure 3:**
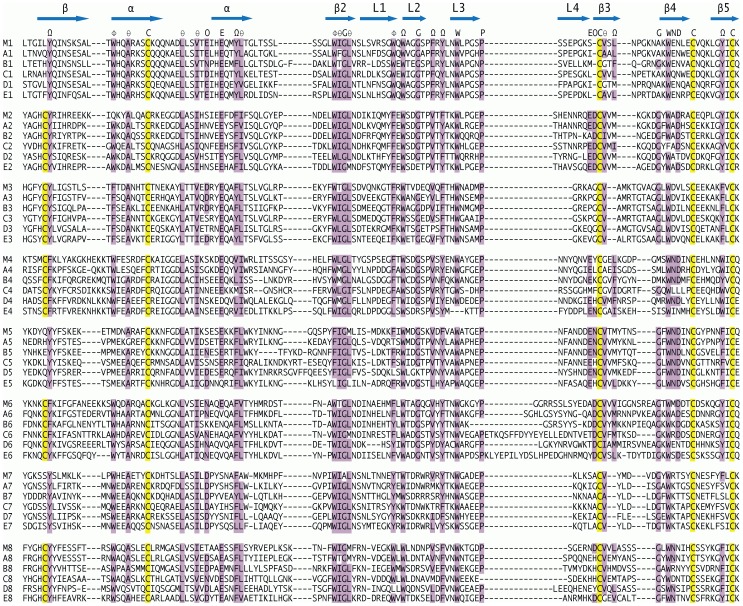
C type lectin domains of the avian MRC1 orthologue gene products. Sequences are labelled on the left, M being the mouse MRC1 sequence while the chicken genes are labelled A to E in genome order in the direction of their transcription, with sequential numbers to indicate the domains in order. Dashes indicate missing residues in the alignment. The short linker peptides between domains are omitted from this figure. Residues reported [20], [51] to be conserved throughout the mannose receptor family are indicated above the sequences using the symbols Ω, aromatic or aliphatic; φ, aromatic; θ, aliphatic; C, E, G, P, W, N, D the standard amino acid codes; O, carbonyl oxygen containing (DNEQ). The corresponding residues in the sequences are shaded, yellow for cysteine and purple for the others. Additional cysteine residues in domains 2, 3, 4, 6 and 8 are also shaded. Likely locations of secondary structural features in the mouse sequence [52] are indicated by blue arrows above the sequence; β, beta strand; α alpha helix; L loop.

The locations of conserved features in the CTLDs of the chicken MRC1L genes are shown in figure 3. The tryptophan/hydrophobic/glycine/hydrophobic (WIGL) motif characteristic of the family [20] is present in all these domains of all genes, with minor variations. Four cysteine residues are also conserved in all these domains, an outer pair forming the disulphide bond spanning most of the domain, and an inner pair forming the disulphide bond stabilising the β3–β4 hairpin [21].

### MRC1L genes in other species

**Figure 4:**
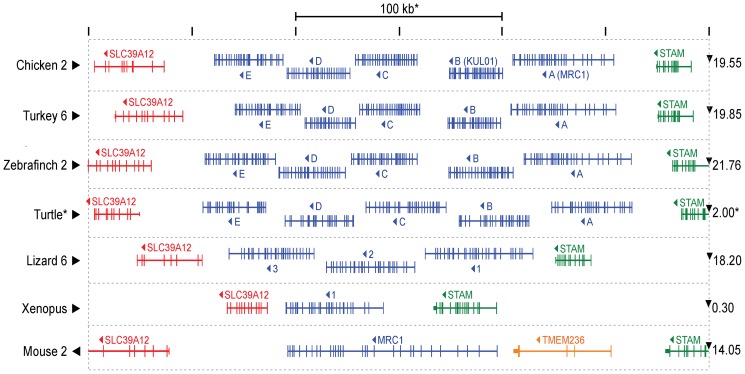
Arrangement the MRC1 orthologue locus in different species. Species are labelled at the left, with a numeral indicating the chromosome where that is known. Black arrowheads indicate the relative orientations of the reference genome maps. The conserved flanking genes SLC39A12 and STAM are indicated in red and green respectively. An additional gene TMEM236, found only in mammalian genomes, is coloured yellow. Predicted MRC1 paralogues are shown in blue. Vertical lines represent the exons of each gene. All the genomes are represented at the same scale, so that the region between vertical dotted lines is 300 kilobase pairs, except in the case of the Painted Turtle, where it represents 600 kilobase pairs. The location in megabase pairs of the right hand end of the map in the chromosome, or other map segment, is indicated at the right. The coding sequences of all genes shown run from right to left in this map, as indicated by arrowheads.

The UCSC genome browser BLAT search [22], [23] with individual and concatenated chicken genes was used to locate orthologous genes in genomes of other birds (turkey and zebrafinch), painted turtle, lizard and Xenopus (sequences in figure S4 and locations in figure S2). In all cases, the alignments with the highest scoring similarities were found between a pair of highly conserved orthologues of the same flanking genes, *SLC39A12* and *STAM*. Exons missing from these BLAT alignments were easily identified by manual inspection. The arrangements of these genes are compared with the orthologous region of the mouse genome in figure 4. The genes in the three birds are very similar, in structure and size of all five genes. The two gaps between the coding sequences of genes C, D and E are small compared with those between the upstream genes. The turtle appears to have a very similar set of five genes, although they occupy a segment of genome twice the length of that in the birds. The lizard genome contains only three genes, while the Xenopus genome, like the mammalian, contains only one *MRC1L* gene. The mouse genome, like that of other mammals, contains an additional gene, *TMEM236*, between the shared flanking genes.

**Figure 5:**
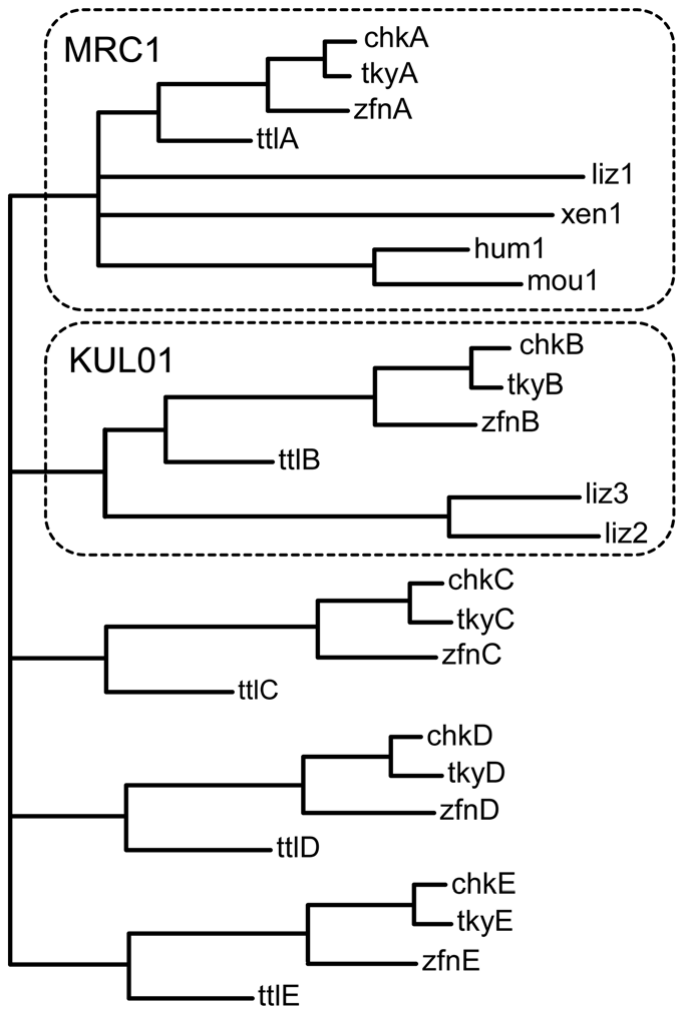
Evolutionary relationships of avian MRC1L genes. A maximum likelihood phylogenetic tree was constructed from predicted exons encoding all the CTLDs, using the Tamura-Nei model in the MEGA software, with 100 bootstrap datasets. All nodes with bootstrap values less than 100 were coalesced into multifurcations. Leaves are labelled with a three letter species code (chk, chicken (*Gallus gallus*); tky, turkey (*Maleagris gallopavo*); zfn, zebrafinch (*Taeniopygia guttata*); ttl, painted turtle (*Chrysemys picta bellii*); liz, lizard (*Anolis carolinensis*); xen, *Xenopus tropicalis*; hum, human (*Homo sapiens*); mou, mouse (*Mus musculus*); followed by either a letter or a number indicating the order of the genes in the direction of transcription. Clades representing orthologues of the MRC1 (human) and KUL01 (chicken) genes are surrounded by dotted lines.

Phylogenetic trees were constructed using a variety of sequence subsets and methods. The great majority of the results had similar topology to the tree depicted in figure 5. Several of the genes identified in other species were missing all or parts of exons in gaps in the genome assemblies. Some signal peptide exons were uncertain, and gene D lacked CysR and FNII domain exons. To avoid bias by these omissions, the tree shown was constructed using just those parts of the CTLDs that were available from all of the genes involved. All species had a single gene that was placed in the same clade as the mammalian *MRC1* gene in 100% of bootstrapped trees. For the avian and turtle genes, the same pattern of species was found for each gene, implying that these arose by duplication before the divergence of these species. In contrast, the lizard lacked orthologues of genes C, D and E, but appeared to have two relatively similar genes of the gene represented in chickens by the KUL01 antigen. Thus the simplest consistent history of this gene family would be an original duplication of the ancestral MRC1 gene, giving rise to the *MRC1L-B* gene, followed in the shared avian and turtle ancestor by further duplications producing genes *C, D* and *E*, and in the lizard lineage by a second duplication of the *MRC1L-B* gene. Trees constructed using all the separated CTLDs generally gave the same pattern of species within a clade representing each domain, providing no evidence for domain reassortment. The majority produced the same topology as the tree shown, although bootstrap values were lower. Where the topologies differed, the bootstrap values were insufficient to support any contrary implications. A minority of alternative tree construction methods failed to place the lizard genes 2 and 3 in the MRC1L-B clade.

**Figure 6:**
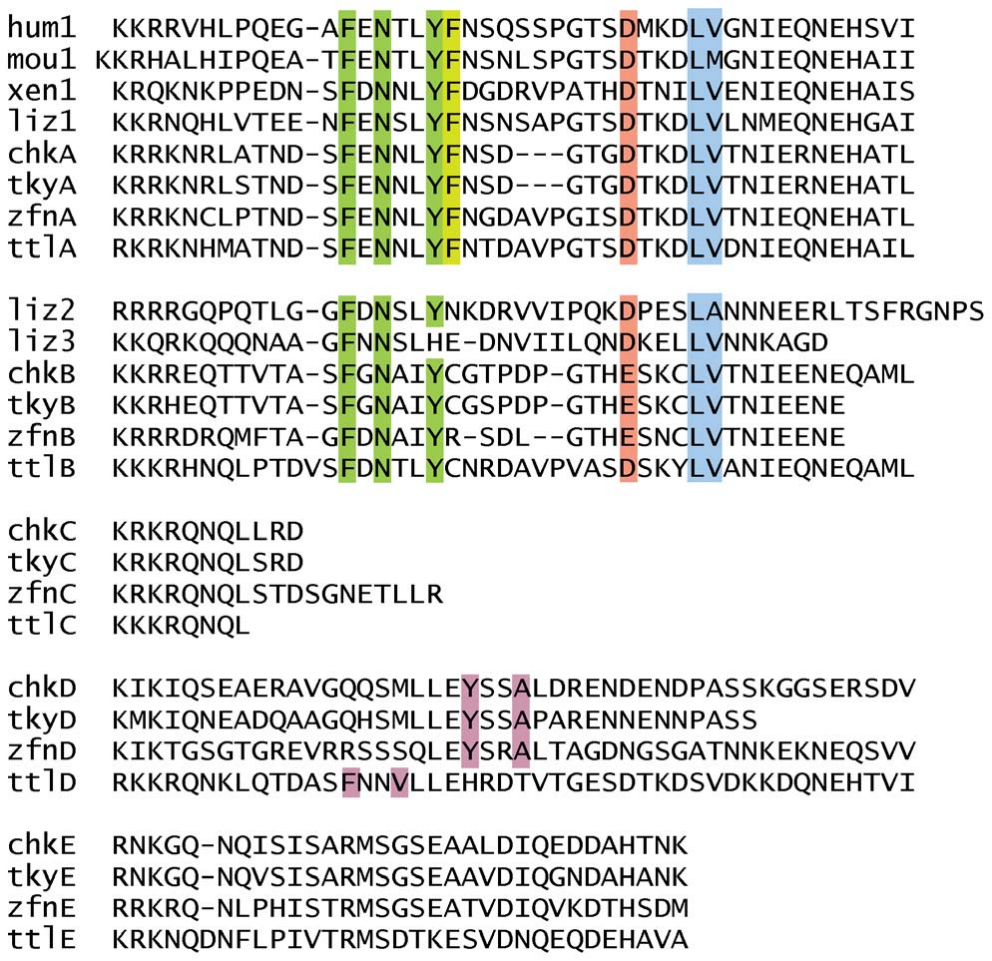
Alignments of cytoplasmic regions of MRC-like genes from various species. Gene names are as described in the legend to figure 5. Shaded residues show the locations of peptide motifs that may be involved in targeting to the endocytic pathway; green for the φxNxxY, red and blue for the (DE)xxxLZ motif, and purple for YxxZ (φ indicating a bulky hydrophobic residue and Z indicating a hydophobic residue). Light green shading indicates an overlapping potential di-aromatic endosome sorting motif in the MRC1 and MRC1L-A sequences.

The cytoplasmic regions of the *MRC1L* gene products are compared in figure 6. The pattern of similarities between sequences are consistent with the evolutionary history that was implied by phylogenetic analysis. This part of the protein is highly conserved between the single mammalian *MRC1* gene and the other genes assigned to the same clade by analysis of the CTLDs. In these molecules, it contains potential motifs involved in targeting to the endocytic pathway, φxNxxY [24], [25] and (DE)xxxLZ [25], [26]. These motifs are shared by the genes that fall into the MRC1L-B clade that includes the KUL01 antigen, except for the replacement of tyrosine by histidine in the second of the two lizard genes in this clade. The group of genes including mammalian MRC1 also has a di-aromatic motif (YF) that may be involved in endosome sorting [27]. Although the latter is absent from the MRC1L-B orthologues, there are several other residues conserved between these two groups of proteins. In contrast, the cytoplasmic regions of the three downstream genes, found only in the bird and turtle genomes, are highly divergent between paralogues, although well conserved among orthologues. The product of MRC1L-C has only very short cytoplasmic sequences beyond the positively charged region expected to lie immediately inside the plasma membrane. Products of genes D and E have cytoplasmic sequences quite different from each other as well as from those of the MRC1L-A and MRC1L-B molecules. None of the downstream genes contain the endocytosis motifs conserved in the two upstream genes, although the MRC1L-D genes do have a potential alternative endocytic pathway targeting motif YxxZ (FxxZ in the turtle) [28].

### Transcription in tissues

**Figure 7:**
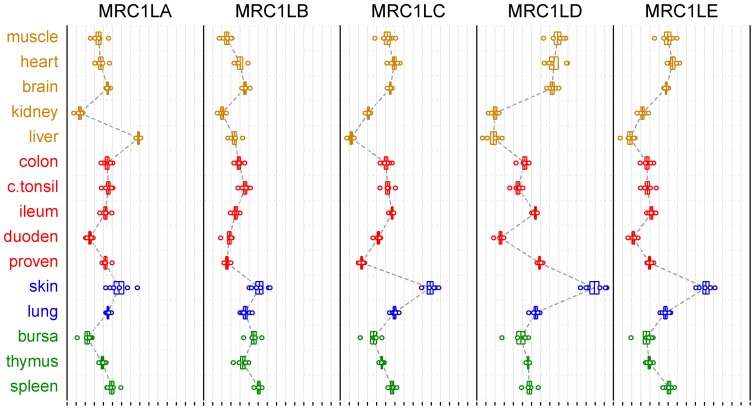
Relative levels of each MRC1 orthologue mRNA in different tissues, measured by quantitative PCR. Tissues, as labelled at the left, are grouped according to preponderance of immune function. For each gene, relative levels of mRNAs are plotted horizontally using a logarithmic scale with arbitrary origins. Circles are individual measurements from each of six birds. Boxes are centered on the means, and their ends indicate the standard errors of those means. All measurements were normalised relative to a constant level of 28S rRNA in each sample and adjusted to the log2 scale using the measured PCR efficiency of standard dilution series, before calculation of means and standard errors. Grey vertical lines and small scale bars at the bottom indicate two-fold differences in relative mRNA measurements.

Amplification of the spliced cDNAs for all five genes from the HD11 cell line suggested that all five genes might be transcribed in macrophages. PCR products were also obtained for all the genes from a spleen cDNA library. To obtain a more general picture of the pattern of transcript levels from these genes, quantitative PCR assays were developed for each and applied to compare levels of mRNA for each gene in different normal tissues. The mRNA levels, relative to 28S rRNA, found in various tissues from six Line 0 birds are shown in figure 7.

Exceptionally, the level of *MRC1L-A* transcript was highest in the liver whilst the level in the skin was highest for the other four genes, though only marginally so for *MRC1L-B*. Genes *C, D* and *E* had remarkably similar patterns of transcript levels in tissues, possibly indicating coordinated regulation. There was some variation between genes in the levels in different parts of the gut, although duodenum always had lower levels than distal regions of the digestive tract. Within the lymphoid tissues the highest level was always seen in the spleen. Relative transcript levels of all the genes were lowest in either kidney or liver. These assays are not calibrated to compare transcript levels between different genes.

The same assay was used to compare levels of expression, relative to 28S RNA, in several transformed cell lines (figure S5). Expression was clearly highest in the two macrophage cell lines, HD11 and MQ. Much lower levels of MRC1L-A were detected in some of the T cell derived cell lines.

## Discussion

In the human genome, the region of chromosome 10 between the flanking markers *SLC39A12* and *STAM* is annotated as containing a tandemly repeated region, each repeat containing the genes *TMEM236* and *MRC1*. The repeated genes are part of a duplicated segment of about 200 kB with greater than 99% identity, separated by a large gap. There are only two BAC end pairs spanning the gap. In contrast all other mammals that we examined, including other primates, have only one copy of the *TMEM236* and *MRC1* genes between the same flanking marker orthologues, without the gap. While a very recent duplication in humans cannot be ruled out, it seems much more likely that this is a mis-assembled region in the human genome and thus that all mammals carry only a single *MRC1* gene. In species from other classes of terrestrial vertebrate, examination of the region of the genomes between the most highly similar homologues of the flanking markers revealed that some of these contained multiple, tandemly arranged diverged paralogues of *MRC1*. *Xenopus tropicalis* genomes contained only a single gene, the lizard *Anolis carolinensis* had three, while three birds and the painted turtle had five. This indicated duplication of the ancestral *MRC1* gene in the avian lineage and its precursors. The most likely sequence of events would have been an initial duplication producing the ancestors of chicken *MRC1L-A* and *MRC1L-B* genes, followed by a much more recent duplication of the latter in the lizard, and by further early duplications in the common ancestor of birds and turtles. In this context, it is of note that the phylogenetic position of the turtle has been the subject of much debate over a number of decades. Whilst a recent report based on an analysis of microRNAs suggested that turtles form a clade with lizards [29], subsequent reports place them in the archosaur lineage with birds and the crocodylia [30], [31]. The more recent proposal is compatible with the simplest possible history of the MRC1 genes described in the present report.

Chicken orthologues of the adjacent *DEC205* [32] and *PLA_2_R* genes, and of the *MRC2* gene, are found elsewhere in the genome. The additional genes in the *MRC1* locus are therefore not relocated orthologues of these genes.

All the identified genes in all the species examined had intact reading frames coding for proteins with the CTLD structure normally found in members of the mannose receptor family. All were found as spliced mRNAs in the chicken. Thus it is unlikely that any of the duplicated genes is a pseudogene, although differently spliced variants of the genes *D* and *E* transcripts were found in HD11 cDNA that had interrupted reading frames. The physical distances between the genes *C, D* and *E* were small, and the pattern of variation of their transcript levels in tissues was very similar. It may be that the transcription of these three genes is co-ordinately regulated by a shared set of upstream *cis*-acting elements. Indeed, the PCR amplifications used to confirm splice junctions would not have detected splicing between exons in different genes, so that the existence of splice variants that combine segments of the three genes, in a manner similar to the TWEPRIL transcripts from the TWEAK-APRIL genes in mouse [33], is not excluded.

The HD11 cell line contained mRNA for all five *MRC1L* genes, but peptides from protein immunoadsorbed by KUL01 included only those from MRC1L-B. This would be consistent with the KUL01 epitope being exclusive to MRC1L-B. However, the similarities between the MRC1L paralogues, while low, are sufficient that we could not exclude the possibility of recognition of the product of one or more of the other genes in the context where KUL01 is applied as a macrophage marker. To test this possibility we conducted two further experiments. As shown in figure S6, treatment of HD11 cells with transfection reagents including a small interfering RNA (siRNA) with 25/25 nucleotide identity to *MRC1L-B* cDNA sequence, caused 90% reduction in the median level of binding of fluorescently labelled KUL01 antibody, compared with the identical levels observed after the same treatment with either a control siRNA without or with no siRNA. The maximum similarity of the effective siRNA with the other *MRC1L* cDNA sequences, in either orientation, were 15/18 (A), 15/23 (C), 16/20 (D) and 17/22 (E). These are similar to the maximum similarity of the control siRNA with MRC1L-B cDNA (16/25), and would not generally be expected to be sufficient for cross-interference. However, since off-target interference effects have been reported with lower similarities, this observation does not completely exclude the possibility of cross reaction with the product of another MRC1L gene. In a second experiment, figure S7, we observed that that the KUL01 antibody only identified MRC1-B when expression plasmids coding for potential extracellular regions of all five MRCIL genes, as fusion proteins, were transfected into COS-7 cells. This provides compelling evidence that the KUL01 anybody binds the product of the MRC1L-B gene and not the remaining paralogues. Whilst the qRT-PCR analysis of MRC1L-B transcripts is consistent with the observed staining patterns reported with KUL01 across a number of immune-related tissues [19] it is not possible from the present data to infer the cellular distribution of the expression of the remaining MRC1L molecules, although, except for MRC1L-A in the liver, the similarity of the transcript profiles would be consistent with their predominant expression in the same cells as MRC1L-B.

In mammals, MRC1 is a multi-functional molecule. Being a pathogen-associated pattern recognition receptor, its involvements in uptake of antigen for presentation are important functions in innate and adaptive immune responses [9], [34], but it also has roles in the clearance of hormones [12] and the regulation of circulating cytokine levels [35]–[37]. Cellular expression of the molecule is not restricted to macrophage alone but is also present on immature dendritic cells, reflecting its role in antigen capture [38].

The information presented here does not tell us whether a shared ancestor of birds and mammals had multiple *MRC1L* genes, with subsequent gene loss in the mammalian lineage, or whether it had a single gene that was subsequently duplicated only in the avian lineage. The former possibility would allow the hypothesis that the modern functions of mammalian MRC1 might have been distributed between the original paralogous genes. The latter model would have allowed the evolution of novel functional roles for the newly duplicated genes. The similarities between the cytoplasmic domains of MRC1L-A and MRC1L-B, especially with regard to trafficking signals, suggest biological functions similar to the mammalian MRC1, with the possibility of functional redundancy between these molecules. The very different cytoplasmic sequences of the other genes might reflect substantial functional divergence of these from the mammalian *MRC1* genes.

The immune functions of MRC1 in the macrophage have given it an important role in determining the effectiveness of the response to influenza virus infection, at least in the lungs of mice. This presents a single interaction that is likely to be an effective target for evolution of viral virulence. If the additional genes in birds have similar functions in avian macrophages, then there is scope for redundant interactions with the virus that might be harder to evade. Expression of all these genes in macrophages is suggestive of conservation of these interactions. It will therefore be important to investigate whether these molecules have suitable carbohydrate binding activities, whether they are involved in endocytosis and phagocytosis, and whether modulation of their expression affects the susceptibility and response to influenza infection of avian macrophages. We have observed abortive replication of influenza in an avian macrophage cell line (KS and CB, Unpublished observations), which would allow a similar protective role for the *MRC1L* genes to that of *MRC1* in the mouse, in generating effective responses. The involvement of multiple molecules, increasing redundancy in virus receptors, could increase the robustness of this immune mechanism in birds.

The known interaction of the mannose receptor with influenza virus in mice allows the hypothesis that a similar situation occurs in birds, facilitating infection of macrophages but leading to a protective innate immune response [18]. There are other enveloped avian viruses, including Marek's Disease Virus, Infectious Bronchitis Virus and Newcastle Disease Virus, that might be supposed to induce IFN-α by interaction with the mannose receptor [14].

Examples in which the mannose receptor acts as an innate pattern recognition molecule include the internalization of the yeast cell-wall particle zymosan [39], the phagocytosis of Pneumocystis by human alveolar macrophages [40] and Mycobacterium tuberculosis by the monocytic human cell line THP-1 [41]. The mannose receptor also appears to play a role in modulating the adaptive immune response through a role in myeloid plasticity [42]. However, the full repertoire of host-pathogen interactions allowed by the mannose receptor, and particularly the relevance of an expanded Mannose Receptor gene family, remains to be elucidated.

## Materials and Methods

### Ethics statement

All animal procedures were performed in accordance with the UK Animals (Scientific Procedures) Act 1986 [43]. This study was approved by the Pirbright Institute Ethical Review Panel and the UK Home Office under project licence 30/2683.

### Experimental animals

RPRL (Regional Poultry Research Laboratory, East Lansing, MI.) Line O birds were obtained from the Compton specific pathogen free breeding facility, from parents negative for antibodies to specified pathogens, and were kept in controlled-environment isolation rooms with food and water provided *ad libitum*. For RNA preparations, tissue sections (approximately 500 mg), from birds between 4 and 5 weeks old, were collected into RNA Later stabilization fluid (Ambion, UK).

### Antibodies, cells and cell lines

KUL01 is a monoclonal IgG1 antibody that recognises an antigen present on the surface of at least a subset of macrophages in chickens [19]. Purified antibody was purchased from Southern Biotech (Alabama).

The retrovirus-transformed macrophage-like cell line HD11 [44] was cultured in RPMI 1640 medium (Invitrogen), 10% FCS. Lines used in figure S5 are described in the figure legend.

### Immunoprecipitation

Five to seven million HD11 cells pelleted at 200×g for 5 min were washed 3 times in PBS and resuspended in 500 µl of ice cold lysis buffer consisting of 20 mM TrisHCl, 100 mM NaCl, 0.5% v/v NP40 pH 7.6 to which 10 µl/ml HALT protease inhibitor cocktail and 10 µl/ml EDTA (Pierce Thermo product 87786) had been added. After vigorous mixing and incubation on ice for 30 minutes, Cell debris was then removed by centrifugation at 17,000×g for 15 minutes at 4°C and the lysate was stored at −80C.

Immunoprecipitations were carried out using a Thermo Scientific Pierce Immunoprecipitation kit (product number 1859011), following the manufacturer's instructions. Three hundred µg of antibody (Southern Biotech) was coupled to 100 µl AminoLink Plus Coupling Resin. Lysates were pre-cleared by two overnight incubations, mixing end over end at 4°C with agarose resin (Thermo Scientific) previously washed in lysis buffer, and then incubated for 2.5 hours with the immobilized antibody. After washing three times with 400 µl lysis buffer, bound proteins were eluted using five 100 µl aliquots of 0.1 M glycine. HCl, pH 2.8 including 0.5% (v/v) NP-40. Proteins were recovered from the pooled eluates by addition of trichloroacetic acid to 10% (w/v) and incubated on ice over night before pelleting in a microfuge at 17,000×g for 20 min at 4°C. Pellets were washed with 1 ml of ice cold 90% (v/v) acetone in water, then dried in a speed vac before resuspension and heating to ≥80°C in PAGE sample buffer including DTT for 10 min. PAGE was performed using 4–12% polyacrylamide Tris-Tricine gels in MES buffer and proteins visualised by rinsing the gels in water then incubating for 1–2 hours in Imperial stain (Thermo Scientific) followed by de-staining in water.

### Peptide analysis

Bands of interest were excised from PAGE gels, cut into 1 mm cubes and individually placed in a covex 96 well microtitre plate. Reduction with DTT, alkylation with iodoacetamide and digestion using trypsin (Promega V511A) were all performed using a Hewlett Packard MassPREP robot. Digested extracts were transferred into low volume glass sample vials (Chromocol), dried in a speedvac then resuspended in 10 µl of 3% acetonitrile with 0.1% TFA. Liquid chromatography was carried out using a Waters NanoAquity UPLC system which supplied solvents A (0.1% formic acid in water) and B (0.1% formic acid in acetonitrile) to a 1.7 µm, 75 µm ×250 mm, BEH 130 C18 column (Waters) (HPLC solvents were all LC-MS grade from Fisher Scientific). Sample was concentrated onto a 180 µm ×20 mm, 5 µm Symmetry C18 trap (Waters) for 3 minutes at 15 µl/min, and separated at 250 nl/min using a gradient which ramped initially from 3–10% B over 1 minute then to 50% B over 41 minutes and to 85% B in 3 minutes followed by a wash step at this concentration for 2 minutes before re-equilibration at 3% B. Ionised peptides were analysed by a quadrupole time of flight (Q-ToF) Premier mass spectrometer (Waters) in data-dependent acquisition mode where a MS survey scan was used to automatically select multicharged peptides for further MS/MS fragmentation. From each survey scan up to four peptides were selected for fragmentation. MS/MS collision energy was dependent on precursor ions mass and charge state. A reference spectrum was collected every 10 seconds from Glu-fibrinopeptide B(785.8426 m/z), introduced *via* a reference sprayer. Raw MS/MS specta were processed using ProtenLynx Global Server (Waters) and were searched against the NCBInr database using the Mascot search algorithm.

### RNA and quantitative PCR

RNA was extracted from 100 mg samples, of fifteen tissues from six birds of the same inbred line, using the Trizol Plus RNA Purification kit (Life Technologies), according to the manufacturer's instructions. Homogenisation was performed using a Mixer Mill MM300 (Retsch) and 3 mm stainless steel cone balls (Retsch). An on-column DNase digestion step was included (Purelink DNAse, Life Technologies). The majority of samples were diluted to have A_260_ approximately 1.0. Some samples with low RNA yields were used at up to ten-fold lower A_260_.

Primers and probes for real-time quantitative PCR assay of 28S rRNA [45] and of the five predicted chicken macrophage mannose receptor mRNAs are detailed in table S3. The *MRC1L* cDNA primers and probes were designed so that the primers were entirely in different exons and the probe was approximately centred on an intron-exon boundary. *MRC1L* gene primers and probes were designed using Genscript primer design software (https://www.genscript.com/ssl-bin/app/primer) and Primer Express (Applied Biosystems, Foster City, California, USA). These primers gave no detectable signal after 40 cycles with 2.5 ng chicken genomic DNA in the standard assay conditions.

Probes incorporated 5-carboxyfluorescein (FAM) at the 5′ end and N,N,N,N′ tetramethyl-6-carboxyrhodamine (TAMRA) at the 3′ end. Assays were carried out using the Superscript III platinum one-step qRT-PCR kit (Invitrogen). Amplification and detection of specific products were carried out with the 7500 Fast Real Time System (Taqman; Applied Biosystems) with the following cycle profile: 50°C for 5 min, 95°C for 2 min and then 40 cycles of 95°C for 3 sec and 60°C for 30sec.

To measure the PCR efficiencies, six 10-fold dilutions, of the highest expressing tissue for each MRC1-L assay, and of HD11 RNA for the 28S assay, were used in triplicate measurements. All *MRC1L* gene mRNA measurements were normalised to the levels of 28S ribosomal RNA in the samples using the equation Xt = Ct−s(Ct′−Q)/s′ where Ct is the gene-specific threshold cycle, Ct′ is the threshold cycle for the 28S ribosomal RNA assay (on a constant dilution of the sample), s and s′ are slopes of linear regressions of threshold cycles (C_T_) against log_10_(RNA) for target gene and 28S assays respectively. All sample Ct values were within the range of the standard plots. Details of the normalisation calculations and of statistical analyses confirming differential expression between tissues are provided in document S1 and document S2.

### Sequencing and bioinformatics

Primers listed in table S4 were designed to amplify overlapping segments of the five predicted transcripts. Preparative PCR amplifications were carried out using methods described elsewhere [32], using templates of a line 0 chicken spleen cDNA library [46], freshly prepared total RNA from line 0 chicken spleen and total RNA from the cell line HD11. Amplified products excised from agarose gels were cloned into the pGEM-T-Easy vector (Promega). DNA prepared using the Qiagen QIAprep spin miniprep kit were used for sequencing. Sequencing reactions were performed by GATC Biotech. Sequence data were analysed using STADEN [47]. Multiple clones of PCR products were sequenced from each amplification to obtain the consensus sequence and to identify clones free from PCR errors.

Extensive use was made of the ClustalW [48]. The UCSC genome browser (http://genome.ucsc.edu; [49]) during the manual refinement of gene structures and in the preparation of figure 4. Assembly versions used were chicken, WUGSC 2.1/galGal3; turkey, TGC Turkey_2.01/melGal1; zebra finch, WUGSC 3.2.4/taeGut1; lizard, Broad AnoCar2.0/anoCar2; painted turtle, v3.0.1/chrPic1; Xenopus tropicalis, JGI 4.2/xenTro3; mouse, GRCm38/mm10; human, GRCh/hq19. Phylogenetic analyses were carried out using the MEGA package [50].

## Supporting Information

Figure S1 Peptide sequences from MRC1L-B found in trypic digest of KUL01-adsorbed material.
(PDF)

Figure S2 Chicken MRC1L cDNA sequences and genomic locations of orthologues.
(FASTA)

Figure S3 Alternative splicing in MRC1L-E cDNA.
(PDF)

Figure S4 Predicted sequences of MRC1L orthologues in other species.
(TXT)

Figure S5 Relative mRNA levels of MRC1L genes in chicken cell lines.
(PDF)

Figure S6 Suppression of KUL01 antigen expression by MRC1L-B specic siRNA.
(PDF)

Figure S7 KUL01 specifically recognises the MRC1L-B gene product in transfected COS cells.
(PDF)

Table S1 List of peptides from tryptyic digest of KUL01-adsorbed material.
(PDF)

Table S2 Differences between Line 0 cDNA sequence and genomic jungle fowl sequence.
(PDF)

Table S3 Primers and probes for TaqMan quantitative PCR.
(PDF)

Table S4 Primers used in amplifying chicken MRC1L cDNAs.
(PDF)

Document S1 Statistical analysis of qPCR data for MRC1L genes in different tissues.
(DOCX)

Document S2 Statistical test for differences in MRC1L transcripts between tissues.
(PDF)

File S1 Chicken MRC1L genes: Links to ENSEMBL identifiers & Errors in release 75.
(RTF)
