# Supplementary material for: Evolution of an Expanded Mannose Receptor Gene Family
Source: PLoS One. 2014 Nov 12;9(11):e110330. doi: 10.1371/journal.pone.0110330 (PMC4229073; doi:10.1371/journal.pone.0110330)
Supplement: Figure S1 — Peptide sequences from MRC1L-B found in trypic digest of KUL01-adsorbed material. (PDF) [file pone.0110330.s001.pdf]

Supplementary figure 1.

```
MAVYLFVIFLFSFCTSLQVSDSGIFLIYNEDSKLCAQAQSSSSVITAMCNENNELQRFRW
ISATQLLSMGMKLCLAVLTQDDGAAITLETCNRTSKLQRWECKDEALSIQDKDLFLNYGT
GKGKKIRLSKESGKGSQWKIHGTAGSICSRPYEDIYTLGGNNFGAPCVFPFKFNDKWFAE
CIRKTDDASTLWCATTSDFDKDQRFGNCPLKDTLHKDFWKTNPLTETHYQINSNSLLTWH
QAKRSCQQQNAELLSVTNPHEEMFLLGLTSDLGFDAKLWTGLVRRLDSSWEWTEGSPLRY
LNWAPGNPSVELLKMCGTFQGRNGKWENVACNQKLGYICQKRNSSIVDDSFTVPSGDVKP
VKCPEEWVAYAGHCYRIYRTPKIWKQAQSSCRKEDGDLTSIHNVEEYSFIVSQLGYKPDD
ELWIGLNDFRFQMYFEWSDGTPVTYTKWQQRQPTHTPNKADCIVMNGEDGFWADSTCERK
LGYICKRKPLAEESGEAEVTYPGCQKGWMKHGFYCYSIGQLPATFSEAKLICEENKAHLA
TVRDRYEQAFLTSIIGFKPVKYFWIGLSDMEEQGTFRWAGGDPVIFTHWNMGMPGREPGC
VAMRTGTSAGLWDILNCEEKNLFLCKQLVEGATPPPPLTTPPPPSCPDEWQSIPQSSFCF
KIFQRGREKMQTWIGARDFCRAIGGDLACIHSEEEQKLISSLNKDYRHVSYWMGLNALGS
DGGFTWCDGSPVNFQKWANGEPNNYDGNEKCGVFYGYNDMKWNDMFCEHMQDYVCQIKKG
ATLKPEPTSTFDYEYIVSEDDWIIYNHKEYYFSKEEMPMEKAREYCKKNGGDLAIIENES
ERTFLWKYTFYKDRGNNFFIGLTVSLDKTFRWIDGSTVNYVAWAPNEPNFANNDENCVVM
YTQTGTWNDLNCGSVELFICERLNRTVRPSIAPTVPPPKGGCPEDWLLFDNKCFKAFGLN
ENYTLTWHAARNNCITSGGNLATISKKENQAFLMSLLKNTATDAWIGLNDINHEHTYLWT
DGSPVYYTNWAKGSRSYYSKDDCVYMKKNPIEQAGKWKDGDCKASKSYICQKNTDPKLQS
SQAVVPMFGFNNYDDDRYAVINYKMNWEEAQKNCKDQHADLASILDPYVEAYLWLQTLKH
GEPVWIGLNSNTTHGLYMWSDRRRSRYHNWASGEPNKNAACAYLDLDGFWKTTSCNETFL
SLCKQFDELIPTESPQLPGKCPEPKQGRSWIPFRGHCYYVHTTSEASWPAASMMCIQMGA
SLVSIEDPAEMNFLLLYLSPFASDNRKFWIGLFKNIEGEWMWSDRSVVEFVNWEKGEPTV
MYDKHCVHMDVSSGAWRNYYCSVDRNFICKIPKIIEVEPTRDSSVSQASKREAAVSSSHS
RAVMIVTLIFLLLAGTGLIVYFFYKKRREQTTVTASFGNAIYCGTPDPGTHESKCLVTNI
EENEQAML
```

Supplementary figure 1. Peptide sequences (grey shading) from the molecule(s) recognized following tryptic digestion and HPLC-MS of protein(s) recognized by the monoclonal antibody KUL01. These are shown at their locations in the sequence encoded by the chicken orthologue of MRC1 that we have called MRCL-B. Some of the shaded segments span overlapping or contiguous peptides. There is one amino acid difference (shaded red) between the genomic sequence (red jungle fowl) and the cDNA, in which pehnylalanine is replaced by serine.
