# Supplementary material for: Evolution of an Expanded Mannose Receptor Gene Family
Source: PLoS One. 2014 Nov 12;9(11):e110330. doi: 10.1371/journal.pone.0110330 (PMC4229073; doi:10.1371/journal.pone.0110330)
Supplement: Figure S3 — Alternative splicing in MRC1L-E cDNA. (PDF) [file pone.0110330.s003.pdf]

Supplementary figure 3. Alternative splicing in MRC1L-E cDNA.

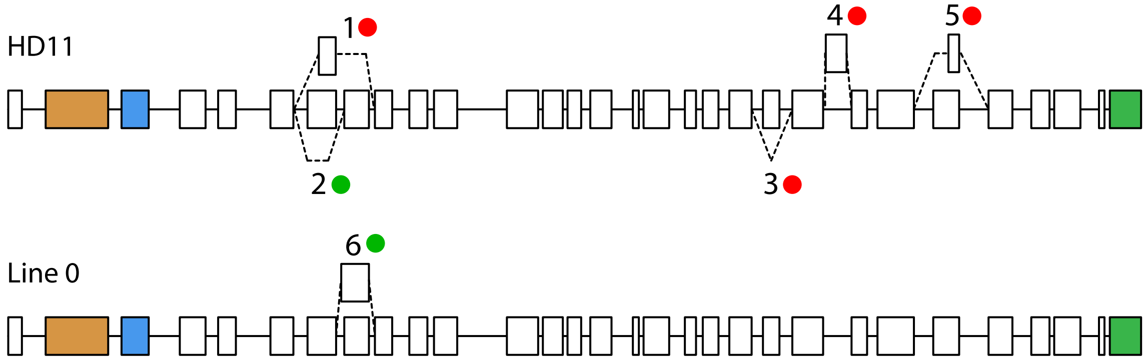

Supplementary figure S3. Alternative splicing in MRC1L-E cDNA in HD11 (top) and in Line 0 (bottom). The central bars represent the major spliced cDNA, as shown in figure 2. Dotted lines link alternatively spliced elements found in one or more cDNA clones. All these used canonical splice donor and acceptor sites. Red dots show which alternative splices caused frameshifts resulting in premature termination of the encoded petide sequence. Green dots show those that retain an intact reading frame.
