# Supplementary material for: Evolution of an Expanded Mannose Receptor Gene Family
Source: PLoS One. 2014 Nov 12;9(11):e110330. doi: 10.1371/journal.pone.0110330 (PMC4229073; doi:10.1371/journal.pone.0110330)
Supplement: Figure S5 — Relative mRNA levels of MRC1L genes in chicken cell lines. (PDF) [file pone.0110330.s005.pdf]

Supplementary figure 5

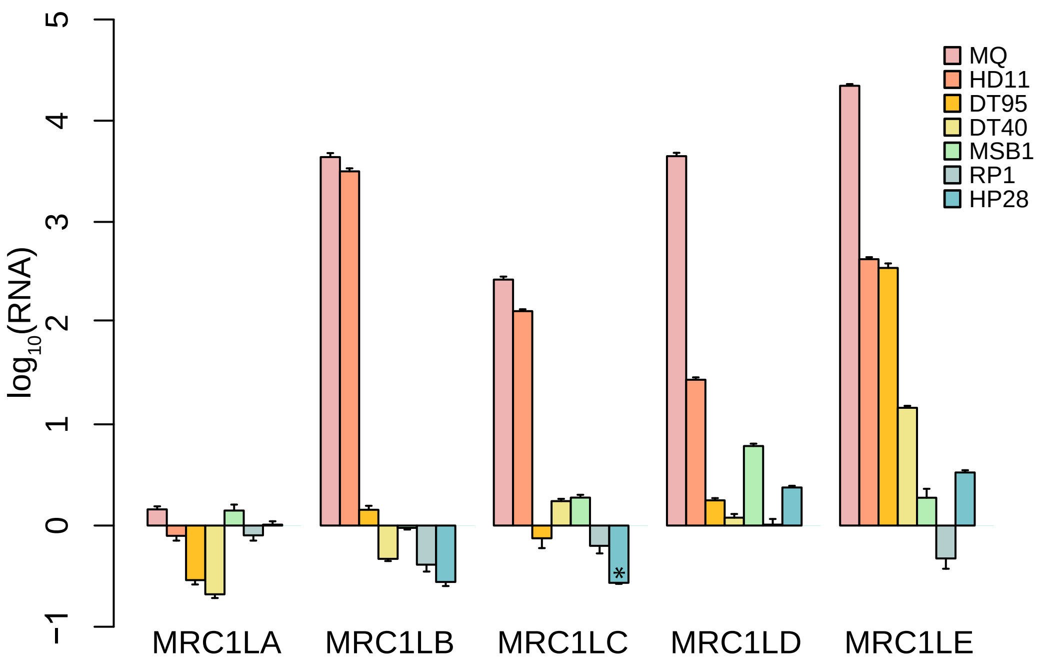

Supplementary figure 5. Relative mRNA levels of MRC1L genes in chicken cell lines.

Bars represent the mRNA levels, normalised to 28S rRNA content, relative to the level measured in the DF1 fibroblast cell line, on a logrithmic (base 10) scale. DF-1 is a spontaneously tranformed chicken fibroblast cell line (Himly et al., 1998). MQ (MQ-NCSU) is a macrophage cell line obtained from a chicken infected with Marek's disease virus (MDV) (Qreshi et al. 1990). HD11 is a retrovirus-transformed macrophage cell line (Beug et al. 1979). DT95 and DT40 are bursal lymphoma cell lines (Baba et al., 1985). MSB-1 (Nazerian and Witter, 1975), RP1 (Nazerian et al, 1977) and HP28 (Payne et al., 1081) are MDV-transformed T cell lines. RNA from Madin-Darby canine kidney (MDCK) epithelial cell line was used as a negative control. DF-1 was grown in DMEM with 10% fetal calf serum and 1mM sodium pyruvate. DT 40 and DT95 were grown in DMEM with 8% FBS and 2% chicken serum. All other lines were grown in RPMI with 10% fetal bovine serum (FBS). All lines were grown at 41°C in humidified 5% CO2. Triplicate RNA extractions were performed using the Purelink RNA mini extraction kit with on-column Purelink Dnase (life technologies). Values plotted are the means of the three replicates. Error bars show the standard errors (n=3, except for the bar with an asterisk where one outlier was removed).

References

Baba TW, Giroir BP and Humphries EH (1985) Cell lines derived from avian lymphomas exhibit two distinct phenotypes. *Virology* 144, 139-151.

Beug H, von Kirchbach A, Doderlein G, Conscience JF, and Graf T (1979) Chicken hematopoietic cells transformed by seven strains of defective avian leukemia viruses display three distinct phenotypes of differentiation. *Cell* 18, 375-390.

Qureshi MA, Miller L, Lillehoj HS and Ficken MD (1990) Establishment and characterization of a chicken mononuclear cell line. *Vet. Immunol. Immunopath.* 26, 237-250.

Himly M, Foster DN, Bottoli I, Iacovoni JS and Vogt PK (1998) The DF-1 chicken fibroblast cell line: transformation induced by diverse oncogenes and cell death resulting from infection by avian leukosis viruses. *Virology.* 248, 295-304.

Nazerian K and Witter RL (1975) Properties of a chicken lymphoblastoid cell line from Marek's disease tumor. *J. Natl. Cancer Inst.* 54, 453-458.

Nazerian K, Stephens EA, Sharma JM, Lee LF, Gailitis M and Witter RL (1977) A Nonproducer T Lymphoblastoid Cell Line from Marek's Disease Transplantable Tumor (JMV). *Avian Diseases* 21, 69-76.

Payne LN, Howes K, Rennie M, Bumstead JM and Kidd AW (1981) Use of an agar culture technique for establish-
