# Supplementary material for: Evolution of an Expanded Mannose Receptor Gene Family
Source: PLoS One. 2014 Nov 12;9(11):e110330. doi: 10.1371/journal.pone.0110330 (PMC4229073; doi:10.1371/journal.pone.0110330)
Supplement: Figure S6 — Suppression of KUL01 antigen expression by MRC1L-B specic siRNA. (PDF) [file pone.0110330.s006.pdf]

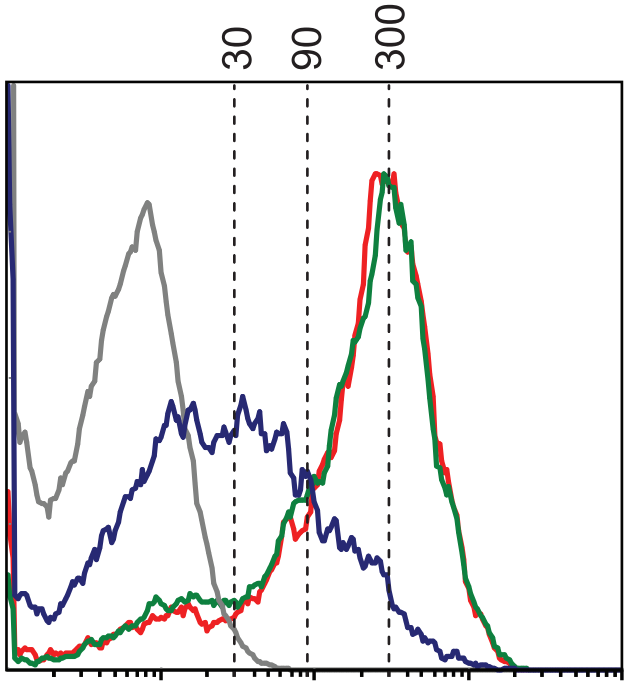

Figure S6. Suppression of KUL01 antigen expression by MRC1L-B specific siRNA. The histograms show the distribution of fluorescence intensity of populations of HD11 cells binding fluorochrome labelled KUL01 antibody. Red is HD11 transfection control without siRNA; Blue is HD11 transfected with the MRC1L-B siRNA-1369 (CCTAATAAGGCAGACTGCATTGTTA); Green is HD11 transfected with the control siRNA-421sc (CATTCAAGACGGACGATACTGGGTT); Grey is HD11 untransfected and unstained. Numbers at the top indicate the relative fluorescence intensity represented by the dashed lines.
