# Supplementary material for: Evolution of an Expanded Mannose Receptor Gene Family
Source: PLoS One. 2014 Nov 12;9(11):e110330. doi: 10.1371/journal.pone.0110330 (PMC4229073; doi:10.1371/journal.pone.0110330)
Supplement: Figure S7 — KUL01 specifically recognises the MRC1L-B gene product in transfected COS cells. (PDF) [file pone.0110330.s007.pdf]

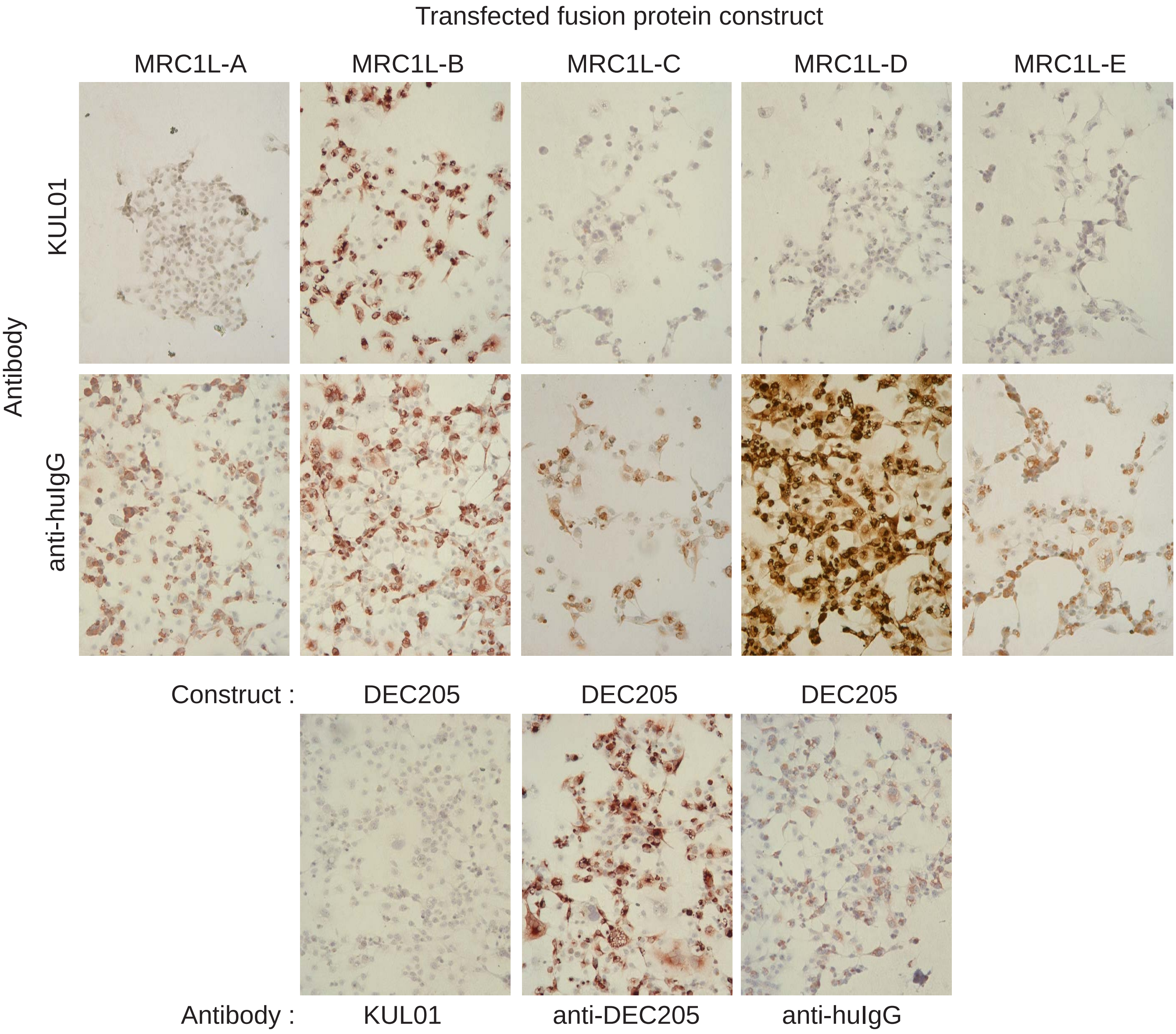

Figure S7. KUL01 antibody specifically recognises the MRC1L-B gene product. Expression plasmids each carrying fusions of sythetic DNA encoding the extracellular domains of one of the five MRC1L molecules, or of the related DEC205 molecule, with human IgG1Fc, were tranfected into COS-7 cells. After 40 hours, the cells were fixed and stained with the indicated antibodies, using corresponding HRP-conjugated secondary antibodies. Cells were counterstained with Haematoxylin and observed by transmitted light microscopy.

The fusions proteins consisted of the peptides comprising the signal sequences and all but the last 7-8 residues of the extracellular domains of each MTRC1L gene product, fused to human IgG1Fc in which the hinge cysteines were replaced by serine residues (Linsley PS et al. [1991] *J.Exp.Med.* 173, 721). The following table gives the junction sequences, with 9 residues from each parental sequence and the linking GR, the UniProtKB accession number of the MRC1L peptide sequence, and the number in that database of the last residue included (underlined).

```
   --MRC1L->  <--huIgG1 [Acc.No seq    ]
A: AMTKDEEATGRDLEPKSSDK [M1X8W0 1-1387]
B: SQASKREAAGRDLEPKSSDK [M1XGZ4 1-1374]
C: KDKQRNEDTGRDLEPKSSDK [M1XH40 1-1376]
D: AVANVKGGRGRDLEPKSSDK [M1XJF4 1-1180]
E: SPENMGQKTGRDLEPKSSDK [M1XGM6 1-1379]
```
