# Supplementary material for: Evolution of an Expanded Mannose Receptor Gene Family
Source: PLoS One. 2014 Nov 12;9(11):e110330. doi: 10.1371/journal.pone.0110330 (PMC4229073; doi:10.1371/journal.pone.0110330)
Supplement: Table S1 — List of peptides from tryptyic digest of KUL01-adsorbed material. (PDF) [file pone.0110330.s008.pdf]

Supplementary table S1. Tryptic peptides from 180kDa protein

Peptide	Start position[1]	End position[1]
LCAQAQSSSSVITAMCNENNELQR	18	41
WISATQLLSMGMK	44	56
WECKDEALSIQDK	84	96
DLFLNYGTGK	97	106
FNDKWFAECIR	157	167
WFAECIR	161	167
TDDASTLWCATTSDFDKDQR	169	188
FGNCPLKDTLHK	189	200
LWTGLVR	262	268
RLDSSWEWTEGSPLR	269	283
LDSSWEWTEGSPLR	270	283
YLNWAPGNPSVELLK	284	298
WENVACNQK	310	318
CPEEWVAYAGHCYR	347	360
FQMYFEWSDGTPVTYTK	415	431
ADCIVMNGEDGFWADSTCER	444	463
KPLAEESGEAEVTYPGCQK	472	490
YEQAFLTSIIGFKPVK	530	545
YFWIGLSDMEEQGTFR	545	561
TGTSAGLWDILNCEEK	589	604
NLFLCK	605	610
MQTWIGAR	654	661
AIGGDLACIHSEEEQK	666	681
WANGEPNNYDGNEK	721	734
CGVFYGYNDMK	735	745
EYYFSK	793	798
NGGDLAIIENESER	813	826
TFLWK	827	831
GGCPEDWLLFDNK	924	936
ENQAFLMSLLK	972	982
YHNWASGEPNK	1151	1161
SWIPFR	1213	1218
FWIGLFK	1272	1278
NIEGEWMWSDR	1279	1289
SVVEFVNWEK	1290	1299
GEPTVMYDK	1300	1308
NYYCSVDR	1322	1329
EQTTVTASFGNAIYCGTPDPGTHESK	1393	1418

[1]Positions are amino acid residues from the N-terminus of the mature peptide.
