# Supplementary material for: Evolution of an Expanded Mannose Receptor Gene Family
Source: PLoS One. 2014 Nov 12;9(11):e110330. doi: 10.1371/journal.pone.0110330 (PMC4229073; doi:10.1371/journal.pone.0110330)
Supplement: Table S2 — Differences between Line 0 cDNA sequence and genomic jungle fowl sequence. (PDF) [file pone.0110330.s009.pdf]

Supplementary table S2. Differences between Line 0 cDNA sequence and genomic jungle fowl sequences.

MRC1L-A		MRC1L-B		MRC1L-C		MRC1L-D		MRC1L-E	
Nucleotide	Peptide	Nucleotide	Peptide	Nucleotide	Peptide	Nucleotide	Peptide	Nucleotide	Peptide
C1107T	-	T2067C	-	390*del*AAG	130*del*K	T504A	-	G63A	-
C1505T	T502M	C2478T	-	A606G	-	A594G	-	A252C	E84D
A1628T	Q543L	C3363T	-	T2916C	-	A609T	-	G417A	-
A2262G	-	C3617T	S1206F	T2931C	-	T738C	-	A408G	-
G4270T	A1424S			C2948T	A983V	A1062G	-	A591G	-
A4290G	V1442D			T2958C	D1037N	C2026T	-	T2120C	L707P
				G3109A	Y1038F	C2166T	-	G2238A	-
				A3113T	-	A3258G	-	G2694A	-
				A3403G	I1135V			A2764G	R922G
				G3465C	K1155N			A2842G	K948E
								A3016G	I1006V
								A3053G	N1018S
								A3055G	T1019A
								C3176G	T1059R
								C3252T	-
								C3546T	-
								C4213G	L1405V
								T4317C	-

Supplementary table S2. Differences between Line 0 cDNA sequence and genomic jungle fowl sequences. Numbers refer to the positions in the precursor protein sequences from Line 0 cDNA, including the signal peptide, as submitted to the ENA database. The residue symbol before the number is from the Line 0 cDNA sequence, and the following symbol is from the red jungle fowl sequence. For the single case of a deletion (*del*), the indicated Line 0 residues are deleted in the red jungle fowl sequence immediately following the position indicated.
