# Supplementary material for: Evolution of an Expanded Mannose Receptor Gene Family
Source: PLoS One. 2014 Nov 12;9(11):e110330. doi: 10.1371/journal.pone.0110330 (PMC4229073; doi:10.1371/journal.pone.0110330)
Supplement: Table S3 — Primers and probes for TaqMan quantitative PCR. (PDF) [file pone.0110330.s010.pdf]

Supplementary table S3. Primer and probe sequences for TaqMan quantitative PCR.

Gene	Type	Sequence	Exons
chkA	Forward	5’- AACCTGGCCACTATCCCTAATG -3’	21
	Reverse	5’- TTAATCCGATCCACGTGTCAGTA -3’	22
	Probe	5’(FAM)- AAGTGCAAGCTTTCCTCACCTTCCATATGA -(TAMRA)-3’	21/22
chkB	Forward	5’-GGAACTGCAGGCAGCATATGT-3’	2
	Reverse	5’-CACAAGGTGCACCAAAATTATTTC -3’	3
	Probe	5’-(FAM)-CAGGCCCTATGAGGATA-(TAMRA)-3’	2,3
chkC	Forward	5’-GTGGAAATGACTGCACCATCTG -3’	28
	Reverse	5’-CATTCCGTTGCTTGTCTTTCTGTA -3’	29
	Probe	5’(FAM)-TCCCACTTGGTTCAACTTTGGGTTTCTTG -(TAMRA)-3’	28/29
chkD	Forward	5’- CTCATCGCGGTCTCCTGAA -3’	7
	Reverse	5’- AGGTTGCAAGTGCAGAACCC -3’	8
	Probe	5’-(FAM)- CCAGCCTTCCTGGCAGCCTGG -(TAMRA)-3’	7/8
chkE	Forward	5’-TGACAGCCTCCATCCAGAAGA -3’	22
	Reverse	5’-GACTCATCGCTCCATTTCCCTATA -3’	23
	Probe	5’-(FAM)-CACAGTCATACTGCATCCGATTGTGGC -(TAMRA)-3’	22/23
28S	Forward	5’-GGCGAAGCCAGAGGAAACT-3’	
	Reverse	5’-GACGACCGATTTGCACGTC-3’	
	Probe	5’-(Yakima yellow)-AGGACCGCTACGGACCTCCACCA-(TAMRA)-3’	
