# Supplementary material for: Evolution of an Expanded Mannose Receptor Gene Family
Source: PLoS One. 2014 Nov 12;9(11):e110330. doi: 10.1371/journal.pone.0110330 (PMC4229073; doi:10.1371/journal.pone.0110330)
Supplement: Table S4 — Primers used in amplifying chicken MRC1L cDNAs. (PDF) [file pone.0110330.s011.pdf]

Supplementary table S4. Primers used for determining the chicken MMR sequences

Name	sequence	Orientation
63-Q-For	5'- ATCACAAGCGCTGTGTTTTG	Forward
63-C-For	5'- CTGCAGAGACTGGTGTGC	Forward
63-C-Rev	5'- GCACACCAGTCTCTGCAG	Reverse
63-F-Rev	5'- CACATACTTGGGTACAGGAG	Reverse
63-E-For	5'- AGGTGCAAGTTTAAAGCCTG	Forward
63-G-For	5'- CAGATGCTGAGTTTCTCCC	Forward
63-H-Rev	5'- CGTCCATTTGTCACATTACTG	Reverse
63-P-Rev	5'- CAAGGCACATTACATTACATTTTACA	Reverse
L-A-For	5'- ATGGCTGTTTACCTTTTTGTG	Forward
L-C-For	5'- CCTCAGGAGACGTGAAGC	Forward
L-D-Rev	5'- AGCCCAGAACCCATCCTC	Reverse
L-E-For	5'- AAGGAGCAACACTGAAACCA	Forward
L-F-Rev	5'- AATGTATTCATAATCAAATGTGG	Reverse
L-G-For	5'- ACTGACCCTAAATTGCAAAGC	Forward
L-H-Rev	5'- GGCCATGAGTCGTGTTGC	Reverse
L-K-Rev	5'- TACTAAAGCATTGCCTGTTCA	Reverse
59-A-For	5'- ATGAGGTTCTATGGGTTCTG	Forward
59-C-For	5'- TCAGGTGACATTTGGCCTA	Forward
59-D-Rev	5'- GCAAAATAGCCATCCTGGC	Reverse
59-E-For	5'- GGAGTGATGCCAAAACCTG	Forward
59-F-Rev	5'- TGGTTTTATACTCATATGTGTC	Reverse
59-G-For	5'- GCCAAATGAATTCATTTACTGC	Forward
59-I-Rev	5'- CAGAAGAGCAATCAGTGTTG	Reverse
59-L-For	5'- ACCAGTGGTTGTGGTTGGAT	Forward
59-M-Rev	5'- TCAATCTCTCAATAGTTGGTTTTG	Reverse
59-N-Rev	5'- CTTTTGCCACAGAACCCATT	Reverse
94-A-For	5'- CATTCAGAAGGCTTTTATGCAA	Forward
94-C-For	5'- GGCTACTGGGCAACTGATGT	Forward
94-D-Rev	5'- AGCCTGGGTCCTTGATCTTT	Reverse
94-E-Rev	5'- CTCCGTGCTTCTTCCATAGG	Reverse
94-G-for	5'- CATTCCACAACTGCTCCAGA	Forward
94-I-Rev	5'- CACGTCACTGCGTTCACTTC	Reverse
94-J-Rev	5'- TATTGTATGCCGGTGCATGT	Reverse
94-K-For	5'- AATGCGCTGGAATGACTTGT	Forward
94-L-Rev	5'- TTCCCCACTGAGCTCTAGGA	Reverse
94-E-For	5'- CCTATGGAAGAAGCACGGAG	Forward
94-G-Rev	5'- TCTGGAGCAGTTGTGGAATG	Reverse
82-Q-For	5'- CTTCCCTTTCCAGCTGAGTG	Forward
83-D-Rev	5'- GAGAGGCGATCTGTTGCTGT	Reverse
82-D-For	5'- ACAGCAACAGATCGCCTCTC	Forward
82-F-For	5'- TGACCAGCTTTGTTGGTCTG	Forward
82-G-Rev	5'- AGGCATTGCTGAGTTCCAGT	Reverse
82-H-For	5'- TGGAATGATGTTAGCTGTGGTC	Forward
82-I-Rev	5'- TCCGGGAGGTGAAGTTACTG	Reverse
82-J-For	5'- TCGTTGGATTGACAGATGGA	Forward
82-K-Rev	5'- CATCTGTCTTCTTACAGATGGAAA	Reverse
82-M-Rev	5'- AAAACTGCCGTGAGATTTGA	Reverse
MMRE-Ex1a	5'-ATGAAATACTTGACTTCTGCAATCTT	Forward
MMRE-Ex1b	5'-CAGCAATTAATTTCAGCTCATCT	Forward
