# Supplementary material for: Evolution of an Expanded Mannose Receptor Gene Family
Source: PLoS One. 2014 Nov 12;9(11):e110330. doi: 10.1371/journal.pone.0110330 (PMC4229073; doi:10.1371/journal.pone.0110330)
Supplement: Document S1 — Statistical analysis of qPCR data for MRC1L genes in different tissues. (DOCX) [file pone.0110330.s012.docx]

Statistical analysis of qPCR data for MRC1L genes in different tissues.

Experimental plan:

RNA samples were independently prepared from samples of fifteen tissues from six birds of the same inbred line, using the same isolation technique. The procedure included extraction with guanidine thiocyanate/phenol followed by column purification including an on-column DNAse treatment. Experiments using a commercially available qPCR for detection of genomic DNA showed that, while this treatment could reduce DNA contamination by several orders of magnitude, it did not eliminate it entirely (i.e. reduce it below the level of detection). The concentrations of A260 absorbing material of the majority of samples were adjusted to be approximately 1 mg/ml (2ug RNA per assay). Some samples with low RNA yields (mostly skin) were used at up to ten-fold lower concentrations.

All the MRC1L cDNA probes and primers used here were tested and gave no detectable signal with 20ng chicken genomic DNA added to the standard assay.

Analysis (tissues_stats_lme_tukey_b.pdf):

All MRC1L mRNA measurements were normalised to the levels of 28S ribosomal RNA in the samples using the equation Xt = Ct – s(Ct’ – Q)/s’ where Ct is the gene-specific threshold cycle, Ct’ is the threshold cycle for the 28S ribosomal RNA assay (on a constant dilution of the sample), s and s’ are respectively the slopes of plots of threshold cycle versus RNA concentration for a series of tenfold dilutions, and Q is the median 28S rRNA threshold cycle for all the samples. All sample Ct values were within the range of those from the standard dilution plots. In this series of samples, the second terms (i.e. s(Ct’ – Q)/s’) were all within the range -2 to +2, except for three muscle samples and the skin samples which had low RNA concentration (A260).

Statistical analysis of the normalised measurements was carried out using the nlme and multcomp packages in R. The approach was to treat each gene separately, using linear mixed effect models, to account for possible random effects between birds, so that systematic differences between birds across tissues, could be eliminated*. There were very minor difference between the results with these mixed effects models and those from models without random effects, indicating that there was only a small bird effect.

*It should be noted that, since only one sample was analysed from each bird, the “bird” effect includes any effects of differences in the sampling of individuals as well as any differences in the genetics or physiology of the birds. The variance contribution from these sources is combined.

To test for significant differences between the means of different tissues, the glht function of the multcomp package was used, with Tukey contrasts, which provides p values adjusted for multiple testing. The output is summarised in the tables provided, using the conventional asterisk symbols to indicate adjusted p values for falsely rejecting the (two sided) alternative hypothesis of no difference, between tissues, of mRNA levels relative to 28S RNA.

Between genes (diff_between_genes.pdf):

Comparison between genes, of the patterns of expression across tissues, was carried out by using an analysis of variance with main gene effect to eliminate different sensitivities of the assays for different genes, and a gene:tissue interaction to compare the remaining differences across tissues. This was followed by Tukey’s honest significant difference analysis to obtain significance scores for the interactions. These are summarised in the table provided.
