# Supplementary material for: Evolution of an Expanded Mannose Receptor Gene Family
Source: PLoS One. 2014 Nov 12;9(11):e110330. doi: 10.1371/journal.pone.0110330 (PMC4229073; doi:10.1371/journal.pone.0110330)
Supplement: Document S2 — Statistical test for differences in MRC1L transcripts between tissues. (PDF) [file pone.0110330.s013.pdf]

Statistical test for differential normalised Ct between tissues.

Line 0 birds, 5 weeks, n=6.

MRC1LA

	sp	ct	pr	il	co	br	lu	th	he	mu	du	bu	ki	li	sk
sp									**	***	***	***	***	***	
ct										*	***	***	***	***	*
pr											***	***	***	***	***
il											***	***	***	***	***
co											***	***	***	***	**
br											***	***	***	***	**
lu											***	***	***	***	**
th											**	***	***	***	***
he	**										*	***	***	***	***
mu	***	*										**	***	***	***
du	***	***	***	***	***	***	***	**	*				*	***	***
bu	***	***	***	***	***	***	***	***	***	**				***	***
ki	***	***	***	***	***	***	***	***	***	***	*			***	***
li	***	***	***	***	***	***	***	***	***	***	***	***	***		***
sk		*	***	***	**	**	**	***	***	***	***	***	***	***	

MRC1LB

	ki	mu	pr	du	li	il	th	co	he	br	lu	ct	bu	sp	sk
ki					***	***	***	***	***	***	***	***	***	***	***
mu							***	***	***	***	***	***	***	***	***
pr							***	**	***	***	***	***	***	***	***
du							***	*	**	***	***	***	***	***	***
li	***									*	**	**	***	***	***
il	***										*	*	***	***	***
th	***	***	***	***									**	***	***
co	***	***	**	*									***	***	***
he	***	***	***	**									***	***	***
br	***	***	***	***	*									***	***
lu	***	***	***	***	**	*								***	***
ct	***	***	***	***	**	*								***	***
bu	***	***	***	***	***	***	**	***	***						
sp	***	***	***	***	***	***	***	***	***	***	***	***			
sk	***	***	***	***	***	***	***	***	***	***	***	***			

MRC1LC

	he	lu	il	sp	br	ct	mu	co	th	du	bu	ki	pr	sk	li
he								*	***	***	***	***	***	***	***
lu								*	***	***	***	***	***	***	***
il									**	***	***	***	***	***	***
sp									**	***	***	***	***	***	***
br									*	***	***	***	***	***	***
ct										*	***	***	***	***	***
mu										*	***	***	***	***	***
co	*	*									***	***	***	***	***
th	***	***	**	**	*						*	***	***	***	***
du	***	***	***	***	***	*	*					**	***	***	***
bu	***	***	***	***	***	***	***	***	*				***	***	***
ki	***	***	***	***	***	***	***	***	***	**				***	***
pr	***	***	***	***	***	***	***	***	***	***	***			***	**
sk	***	***	***	***	***	***	***	***	***	***	***	***	***		***
li	***	***	***	***	***	***	***	***	***	***	***	***	**	***	

MRC1LD

	ct	bu	co	th	sp	il	lu	pr	du	li	ki	br	he	mu	sk
ct					*	***	***	***	***	***	***	***	***	***	***
bu						**	***	***	***	***	***	***	***	***	***
co								***	***	***	***	***	***	***	***
th								*	***	***	***	***	***	***	***
sp	*								***	***	***	***	***	***	***
il	***	**							***	***	***	***	***	***	***
lu	***	***							***	***	***	***	***	***	***
pr	***	***	***	*					***	***	***	*	**	***	***
du	***	***	***	***	***	***	***	***				***	***	***	***
li	***	***	***	***	***	***	***	***				***	***	***	***
ki	***	***	***	***	***	***	***	***				***	***	***	***
br	***	***	***	***	***	***	***	*	***	***	***				***
he	***	***	***	***	***	***	***	**	***	***	***				***
mu	***	***	***	***	***	***	***	***	***	***	***				***
sk	***	***	***	***	***	***	***	***	***	***	***	***	***	***	

MRC1LE

	ki	il	ct	co	th	bu	pr	sp	lu	br	he	mu	du	li	sk
ki								***	***	***	***	***	*	***	***
il								***	***	***	***	***	***	***	***
ct								***	***	***	***	***	***	***	***
co								***	***	***	***	***	***	***	***
th								***	***	***	***	***	***	***	***
bu								***	***	***	***	***	***	***	***
pr								***	***	***	***	***	***	***	***
sp	***	***	***	***	***	***	***						***	***	***
lu	***	***	***	***	***	***	***						***	***	***
br	***	***	***	***	***	***	***						***	***	***
he	***	***	***	***	***	***	***						***	***	***
mu	***	***	***	***	***	***	***						***	***	***
du	*	***	***	***	***	***	***	***	***	***	***	***			***
li	***	***	***	***	***	***	***	***	***	***	***	***			***
sk	***	***	***	***	***	***	***	***	***	***	***	***	***	***	

Statistics

Linear mixed effects: lme(ct ~ tissue|bird, tisssues.cor)

{ tissues.cor = normalised Ct }

Test for differences of means: glht(model,linfct=mcp(tissue="Tukey")

P values for non differential expression, adjusted for multiple tyesting.

*** adjusted $p < 0.001$

** adjusted $p < 0.01$

* adjusted $p < 0.05$

R 2.15.2 :Libraries nlme and multcomp.

	n=	tissue		n=	tissue		n=	tissue
br	6	brain	he	6	heart	mu	6	muscle
bu	6	bursa	il	6	ileum	pr	6	proventriculus
ct	6	cecal tonsil	ki	6	kidney	sk	6	skin
co	6	colon	li	6	liver	sp	6	spleen
du	6	duodenum	lu	6	lung	th	6	thymus
