# Supplementary material for: Evolution of an Expanded Mannose Receptor Gene Family
Source: PLoS One. 2014 Nov 12;9(11):e110330. doi: 10.1371/journal.pone.0110330 (PMC4229073; doi:10.1371/journal.pone.0110330)
Supplement: File S1 — Chicken MRC1L genes: Links to ENSEMBL identifiers & Errors in release 75. (RTF) [file pone.0110330.s014.rtf]

Chicken MRC1L genes supplementary data: Links to ENSEMBL identifiers and comments. 

Errors in current ENSGAL transcripts (release 75. Feb 2014).

1. Links
Gene		ENSEMBL gene		ENSEMBL transcrtipt	comment
MRC1LA 	ENSGALG00000008629	ENSGALT00000014063	with errors
MRC1LB	ENSGALG00000028304	ENSGALT00000043091	no errors
MRC1LC	ENSGALG00000008638	ENSGALT00000014059	no errors
MRC1LD	ENSGALG00000027765	ENSGALT00000045580	partial gene with errors
MRC1LD	ENSGALG00000019667	ENSGALT00000014060	partial gene with errors
MRC1LE	ENSGALG00000028357	ENSGALT00000046122	partial gene with errors

2. Enumeration of ENSEMBL errors

Note: exon and intron numbers refer to the "correct" cDNA sequences presented here.

MRC1LA (ENSGALT00000014063)
	ex1		Incorrect signal peptide exon (Exon 1). 
	ex2		Incorrect splice; Non-canonical splice acceptor; 8nt intron 1 in exon 2; 
  phase error.
	in21-ex22	Incorrect splice; Non-canonical splice acceptor (AA); 6nt iinton 21 in eoxn 22.
	ex23-in23-ex24	Incorrectly spliced intron; Non-canonical splice donor (GC); Non-canonical splice 
acceptor (AT); four nt of exon 23 in intron; seven nt of intron in exon 24;     phase error.
	ex29		Split into 2 exons, the last 18nt from the correct exon and the preceding 24nt
  from the intron. The correct exon is 54 nt, ending at thje same point.

MRC1LD is erroneously split into two transcripts...

MRC1LD (ENSGALT00000045580)
	in1		Extraneous exon inserted in intron 1.
	ex16		Splice donor at end of exon 16 missed. Runs into stop codon after translating 19nt of intron.
			Remaining 12 exons missing.

MRC1LD (ENSGALT00000014060)
			First 16 exons missing.
	ex17		Misses splice acceptor at start of exon 17 and assumes translation initiation 
			from nearest in-phase ATG in intron 16, including 53 nt of intronic sequence. 
			Predicted n-terminus does not encode a plausible signal peptide (SignalP 4.0).  

E. MRC1LE (ENSGALT00000046122)
	ex1-2		Exon 1 is missing. The transcript starts in the middle of exon 2.
	ex20		Splice donor at end of exon 20 is missed so that the sequence runs to a stop 
			codon in intron 20 after 6nt of intron sequence.
	ex21-30	Exons 21-30 are missing. There is no ENSEMBL annotation for these exons.
